# A Manual of Psychological Medicine; Including the History, Nosology, Description, Statistics, Diagnosis, Pathology, and Treatment of Insanity

**Published:** 1858-09

**Authors:** 


Art. IV.—*A Manual of Psychological Medicine: Including the History, Nosology, Description, Statistics, Diagnosis, Pathology, and Treatment of Insanity; with an Appendix of Cases.* By John Charles Bucknill, M.D., Superintendent of the Devon County Lunatic Asylum, etc., etc., and Daniel H. Tuke, M.D., Visiting Medical Officer to the York Retreat, etc. etc. Philadelphia: Blanchard & Lea, 1858. 8vo., pp. 536.

It might be supposed that the difficulty, in many cases, of arriving at a correct diagnosis, and of carrying out a successful treatment of insanity, would, continually, stimulate the medical world to a careful and prolonged study of this disease, than which none tries so severely the affective feelings of all who are in any way brought into relation with the sufferer. And yet, by one of those anomalies which every now and then puzzle us, the disordered functions of the brain, constituting insanity, receive less attention in most systematic courses of medical instruction than those of any, even the most ignoble organ of the body. Sometimes the subject is not reached at all by the lecturer; generally, when noticed, it is in a meagre and most unsatisfactory manner; and rarely with any approach to a distinct delineation of pathology and treatment, or to a nicety of appreciation of the blended mental and material agencies which bring on the disease, and still less of those which are to be called into operation for its cure. How many more medical men shall we not find to enter keenly into a disquisition on the several stages of syphilis than of insanity? How many, expert in the diagnosis of pulmonary and cardiac affections, who are mere novices in all that relates to the chronic derangements of the brain? It may be alleged, that insanity is of much less frequent occurrence than syphilis, or than diseases of the lungs and heart; but this is a reason the more for a careful study of the former, so that we may not be taken unawares and unprepared when we have to encounter, and are expected to remove, this sore affliction.

The prevalent neglect of the subject brings with it results injurious both to the insane individual and to the physician: to the former, by his reason being allowed to remain captive for want of suitable knowledge and skill on the part of those who ought to give it freedom; to the latter, by the slur cast on himself and the profession, when he appears as a witness before a court of law, to give his opinion of the real mental condition of an individual, respecting whose sanity doubts may be entertained; or of one, whose insanity it is necessary to be assured, either for the purpose of justifying suitable restraint, or of exempting from penal punishment a person who, as insane, is no longer a free agent. Are practitioners of medicine prepared to appear before a court of law or a commission of lunacy, to give clear and definite answers to the questions that may be propounded to them on these several points? The answer is found in the records of nearly every case, either of real or of alleged insanity, in which physicians appear as witnesses before a judicial tribunal. Ignorance of fundamental principles, contradictions, paradox, and speculation, constitute, too often, the mortifying exhibition on the part of the medical witnesses. The judge can scarcely conceal an ironical smile; the lawyers chuckle; the attending crowd are puzzled at hearing their cherished oracles utter such strange dogmas, which partake neither of the learning of the schools nor of meditation on the subject, nor of personal observation or inquiry; but which are often mere random conjectures of the moment; a jumble of extemporaneous guesses, as if the question of the state of mind of the unfortunate individual were to be determined by a throw of the dice or by a toss of a half-dollar—the draped figure up, he is sane; the eagle up, he is crazy. These strictures are, of course, meant to be applied to the medical profession at large, but without including a fast-increasing body of physicians who have the medical superintendence of asylums for the insane, and who, at their annual conventions, make a free interchange of their experience, and offer suggestions for further improvement.

To prevent such discreditable displays of professional shortcomings, by furnishing a faithful digest of existing knowledge on the subject of insanity, is the object of the work the title of which heads this paper. The authors, in their Preface, say that they have long felt the want of a systematic treatise on this disease, "adapted to the use of students and practitioners of medicine." They very truly assert that "a knowledge of the nature and treatment of insanity is now expected of every well educated man. The India Board require it of all persons to whom medical appointments are given under their new system of competitive examination." While disclaiming the design of making any amount of reading a substitute for clinical knowledge of mental diseases, they state their aim to be "to supply a text-book which may serve as a guide in the acquisition of such knowledge, sufficiently elementary to be adapted to the wants of the student, and sufficiently modern in its views and explicit in its teachings to suffice the demands of the practitioner." With what success this design has been carried out, will in a measure be made to appear more clearly by a brief analytical notice of the work than by any anticipatory opinion of our own. The first part of the volume (to page 267) is by Dr. Tuke; the second by his associate, Dr. Bucknill.

The first chapter consists of a "Historical Sketch of Insanity among the Nations of Antiquity," mainly in regard to its extent. Having stated *in*

*limine*, his conviction that, *other things being equal*, civilization does, on the whole, render men more liable to mental disease, Dr. Tuke next inquires "into the extent to which the great bulk of the people among the ancients were civilized; especially as compared with modern society." The inference is meant to be drawn, that, in proportion as the ancients fell short of the moderns in civilization, so were they less affected with insanity. The Egyptians, the Greeks, and the Romans are passed in review, and measured by this standard; and the author draws the conclusion that they had not reached that extent of civilization which, in the nations of modern Europe, "tends, in some degree, to exhaust and derange the mental powers." But that insanity was known to the ancients is shown by examples, in history, of persons who were insane, or who feigned insanity, as well as by references made to the disease by medical and other writers.

The feigned madness of Ulysses, and the real or supposed madness of Ajax, are referred to, as also the simulated dementia of King David. The graphical description of the terrible insanity of Orestes, by Euripides, is introduced on the present occasion, and also the opinion of Plato on the causes and character of insanity. "It is worthy of remark that, throughout the Mosaic Law, no provision is made which recognizes the possible occurrence of madness." A memorable example of the disease is presented in the person of Cambyses, as we find it described in the pages of Herodotus. To this example may be added that of Cleomenes, King of Sparta, which was attributed to his imitating the Scythians in drinking unmixed liquors. Pythagoras was acquainted with epilepsy. Hippocrates gives a few sketches of insanity, and the description of a case of melancholia; but, "on the whole, they are rather scanty, and would seem to indicate that he had not occasion to treat mental maladies to any very great extent." "The references made incidentally by the Roman poets to insanity are frequent." Many pages might be filled with selections from Ovid, Plautus, Horace, Virgil, and others.

The second chapter is devoted to a review of the opinions of ancient medical writers on the treatment of the insane. Among these, "Asclepiades was certainly one of the most definite in his directions in regard to the treatment of the insane." He it was who prescribed music as a remedy. Cœlius Aurelianus enters with considerable fullness into the treatment of the disease; and, upon the whole, he may be regarded as the best authority among ancient writers on this subject; and as superior even to Galen of Pergamos.

In the third chapter, the subject of "Modern Civilization, in its bearings on Insanity" is discussed. The difficulty of determining the proportion of the insane to the population, whether in regard to various countries, or in regard to the same country at different periods of its history, and the fallacy of various estimates made with this view, are stated in strong terms in the work before us. People have often confounded the knowledge of the evil with the existence of the evil—forgetting that the two things are very different. The proportionate numbers of the insane among some of the nations which we regard as civilized, are represented to be as follows: In France the number has been estimated as one in every 795; in Norway as one in 551; in the Rhenish Provinces as one in 666. In England, as we are told by the author, we cannot doubt that, were the cases not reported to the Lunacy and Poor Law Commissioners fully known, it would, when added to the numbers already ascertained, exhibit, at least, a proportion of one insane (or idiotic) person to every 300 of the population; but, as the figures now stand, it will be said that there is one insane person, or idiot, in 577 of the entire population of England and Wales. In Scotland the proportion is one in 390. Distinguishing the sexes, we find the proportion among males to be one in 616, and among females one in 543. From the results of the census of the insane and the idiotic in Massachusetts, in 1854, it appears that there was at that period one lunatic in every 427, and one idiot in every 1034 of the population; being a proportion, of both, of one in every 302. In the County of Franklin (population 31,735) the proportion was one in 229; and in Duke's County so high as one in 159.

In connection with the question, whether the most frequent causes of insanity are to be found in greater or less force in civilized countries than in those which are not civilized, we should inquire into the influence of an overworked brain, which is proved to be tasked more constantly in the former than in the latter. There is more thought, and more intensity and variety of cerebral excitement in the highly artificial life belonging to civilization, than to the simpler forms of social life of the savage or the uncivilized. "Knowledge brings with it miseries as well as blessings;" and the substitution of great mental for manual labor is too often followed, as in the case of Hugh Miller, the author of the "Old Red Sandstone," by a complete break-down, and insanity in some form or another. There are circumstances almost necessarily associated with excessive study, which are recognized causes of mental disease. "Loss of sleep, by which the nutrition of the cerebral tissue is necessarily interfered with, is one of the most important of these." But, a more powerfully contributing cause of insanity than that of great or excessive and prolonged exercise of the intellectual faculties, is to be sought for in the emotions and passions, which are constantly developed and in strained action in a highly civilized condition of society. Dr. Guislain's remark is quoted on this point: he forcibly observes, that "the people of European civilization and of North American civilization are, as it were, in a state of continual intoxication—

intoxication of the emotions, intoxication of personal dignity, intoxication arising from constantly renewed impressions. It is not thus with those nations which most closely approach a state of nature—men who live far from what we call the tumults of the world." As regards the causes of insanity, under the division of moral and physical, it has been generally thought that the former considerably preponderate. They are most operative among a civilized people; and chiefly among these latter do they display themselves in times of political and religious commotions and revolutions—which have had their share in adding to the number of inmates of lunatic asylums in England, in America, and, above all, as respects political changes, in France. We have ourselves heard Esquirol say, in a lecture at the Salpetriere, that from the first breaking out of the Revolution in 1789, down to the time in which he made the remark, (immediately after the assassination of the Duke of Berri,) no political event or change had occurred without its producing cases of insanity. Dr. Tuke accounts for the apparent anomaly, supposing it to be a fact, of there being a larger number of pauper lunatics in the agricultural than in the manufacturing districts. Drink and want, and consequent domestic suffering, produce their certain effects in the generation of insanity, in the first as well as in the last of these. But the assumption of the greater amount, proportionally, of insanity in agricultural districts is not proved—it is not true. We cannot follow the author in the statistical details which he brings to bear in confirmation of his side of the question. Of the causes which are properly physical, "the most important for consideration is intemperance." The Report of the Commissioners on Lunacy, in 1844, shows "that of 9867 cases in which the causes of insanity was ascertained, 1792, or upwards of eighteen per cent., were caused by the abuse of intoxicating liquors. In America, the proportion is stated to be very much higher among the patients admitted into some of the State asylums. But, we believe that all these figures fall far short of presenting a true picture of the complex influence of intemperance in inducing, directly or indirectly, derangement of the mental faculties. The grand cause of the general paralysis of the insane, Dr. Guislain believes to be the united action of drink and study, or chagrin." Disorders of the uterus are more frequent, and bring with them a longer train of sympathetic nervous and mental disturbances in civilized than in savage life. Among other important physical causes of insanity, intermarriage of blood relations is one. The subject of the comparative frequency of insanity in civilized and in savage life is carried out by Dr. Tuke; but we are unable, with our scant space, to borrow just now either his arguments or his facts, and we are obliged to pass on to the next chapter.

This is on the "Amelioration of the Condition of the Insane in Modern

Times, especially in regard to Mechanical Restraints." The great, and we might almost say wonderful, improvements in this respect, during the present century especially, speak well both for the increasing intelligence and humanity of the people of the present age. The gradual progress made in the right path is agreeably and instructively narrated in the present volume, both in regard to legislative enactments and individual efforts; among the latter of which, as opening the right path, were those made by Pinel in France, in 1792, at the Bicetre Hospital, and by the late William Tuke, in England, in the same year, at the York Retreat.

The "Definition of Insanity, and Classification," constitute the subjects of chapter fifth. The extreme difficulty, not to say impossibility, of giving a correct definition of insanity, one that shall convey unmistakably a clear idea of its varied character, is dwelt upon by Dr. Tuke Not a few difficulties beset, also, the subject of classification. The author, after passing in review those adopted or recommended by different writers and teachers, avows his preference for what he calls "the threefold classification of the mental faculties," the which is that laid down by the phrenologists. It consists of three classes of disorders of the mind: viz., those affecting the intellect, those of the moral sentiments, and those of the propensities—a division which, however, it may be regarded metaphysically, is by far the most natural of any previously offered. Dr. Tuke, notwithstanding, rests satisfied with simply placing before the reader little more than the typical forms of mental disease, with but slight reference to mental analysis, and with none to the pathological conditions with which these forms are doubtless associated.

The "Various forms of Mental Disease" are described in chapter sixth; the first section being on *Idiocy*, *Cretinism*, and *Imbecility*. "In general, our practical knowledge of the characters of mental disorder must, like that of other diseases, be derived from two grand sources—the subjective and the objective. The former is exhibited in what insane persons tell of themselves in their conversation and autobiographies, and is highly instructive; the latter includes the phenomena observed by ourselves as spectators of the disease." Idiocy is a congenital deficiency of the mental powers, which, until the experience of late years has shown what can be done by educating idiots, was believed to be incapable of amelioration or change. There are different degrees of idiocy. In the lower forms "the idiot is below the plant; nutrition is most imperfect, and the power of reproduction null. He would perish but for the assistance of others." In the higher manifestations of function, some idiots possess aptitudes and inclinations; and almost all, even those who are deprived of the power of speech, sing and retain a recollection of tunes. A variety of cases, illustrating the different degrees of idiocy, are introduced by Dr. Tuke. Cretinism, though allied to idiocy, differs from it in some important particulars. The former is acquired, and not congenital, although there is a strong hereditary predisposition to the disease. It is endemic, also, and in this respect differs from idiocy. The cretin is further distinguished by the brown or yellow color of the skin, the remarkably high and arched palate, and, commonly, enlargement of the thyroid gland. Cretinism is more curable than idiocy. In the former there is a defect in the development of all the organs; in the latter the defect is chiefly in the brain. Imbecility is next described, and wherein it differs from idiocy is pointed out. "Idiocy always is, imbecility is not, necessarily congenital; idiocy implies a less amount of intellectual power than imbecility."

*Dementia* is the theme of the second section of this chapter. It is in all cases the result of exhaustion of the mental powers from previous forms of cerebral affections, such as melancholy and mania, or from the natural wearing out of the organ, as in old age. Some, without any previous stage of mental disease, have suddenly, and, it may be, by some overpowering shock to the nervous system, become subjects of dementia. Esquirol, the first to draw the line of distinction clearly between dementia and idiocy, adduced, among other points of difference, the following contrast in their mental resources:—"A man in a state of dementia is deprived of advantages which he formerly enjoyed. He was a rich mán, who has become poor. The idiot, on the contrary, has always been in a state of want and misery." Dr. Pritchard lays down four stages of dementia: viz., forgetfulness or loss of memory, irrationality or loss of reasoning, incomprehension, and inappetency, or loss of instinct and volition. Dementia is sometimes primary, but much more commonly consecutive, as already stated. The acute form of the disease is very rare.

The author next passes, in another section, to the consideration of *Delusional Insanity*—monomania, or partial insanity. The last terms are indicative of the disease. In this part of his work Dr. Tuke restricts it to *intellectual* monomania, while he admits *affective* monomania, and a mania without delirium, or instinctive monomania; which two states he notices subsequently. The most striking illustrations of monomania are found in the mental states called hallucination, illusion, and delusion; words which do not convey to the mind of the uninitiated the sense in which they are employed. We are unable to repeat even a portion of the definitions of and commentaries on them introduced by Dr. Tuke; or to notice many cases which he appositely introduces in elucidation of this very important branch of his subject—one which, in its medico-legal bearings, requires a careful study on the part of every practitioner of medicine. *Melancholia* takes up another (the fourth) section of this chapter. The disease may be simple, complicated, acute, chronic, remittent, or intermittent. The first is represented to be unaccompanied by any disorder of the intellect, strictly speaking—no delusion, no hallucination. It approaches closely to what Dr. Pritchard calls moral insanity. On this point, Dr. Tuke, after some remarks on the prejudice against admitting a man to be insane who is in the possession of his intellectual faculties, says:—"And yet, if it be admitted (and every writer of authority does admit) that a profound melancholy, for which the patient is irresponsible, is not inconsistent with the operations of the intellect, we are called upon to admit no *new* doctrine in mental pathology, when asked to believe that a like condition of the intelligence may exist with a homicidal propensity, in however small a proportion of cases this may actually occur."

But it is more especially in the next section, in which "*Emotional Insanity*" is discussed, that the various forms of moral insanity are brought to the notice of the reader. These are homicidal and suicidal mania, kleptomania, erotomania, pyromania, and dipsomania. They may be complicated with disordered intellect, or they may not; they may be automatic and sudden in their action, or not. Rarely are they, strictly speaking, monomaniacal. The cases introduced by Dr. Tuke to illustrate these several forms, are numerous and full of interest. *Homicidal mania* manifests itself under very different mental conditions. It may or may not be associated with decided lesions of the intellect. It may or may not be impulsive in character. It may or may not be preceded by appreciable premonitory symptoms. It may or may not be manifested from early life. Of *Suicidal mania*, it is said that many remarkable instances are on record of its descending from one generation to another. "It has been observed much more among insane persons who have committed self-destruction than among the sane." In regard to the influence of the seasons, "it is unquestionable that there is the largest number of suicides in spring and summer—a fact which might scarcely have been expected *a priori*." Women more rarely commit suicide than men; and less in the married than in the unmarried state. *Kleptomania*, or the propensity to steal, is sometimes strikingly hereditary. Every community furnishes examples of this form of insanity. The author sarcastically remarks:—"*Kleptomania* is never urged as a defence for the delinquencies of the poor; but when ladies of respectable connection are detected in habits of stealing, the theory of kleptomania has been found exceedingly convenient." On the subject of *Erotomania*, distinctions are pointed out between it and nymphomania and satyriasis—in the first, being a disorder of the imagination; while the two last are the effects of morbid irritation of the organs of reproduction, whose irritation reacts upon the brain. *Erotomania*, in its extended signification, not unfrequently follows upon religious melancholy We pass over *Pyromania*, or the incendiary propensity, as a speculative variety, to notice that other form of emotional insanity called *Dipsomania*, or *mania crapulosa*, or *ebriosa; oinomania*. Dr. Tuke indicates, in treating this subject, the necessity of distinguishing this disease from what may be termed a merely physiological condition, in which the human animal chooses to indulge in alcoholic beverages to excess. We try to establish a diagnosis by observing whether there are any symptoms which can be traced to primary disorders of the nervous system. "The family psychological history, again, is of great importance. Cases in which an insane parent has a drunken son, point strongly, of course, to disease. But the prominent feature of this propensity is its *irresistibility*." The disease appears, in Dr. Hutcheson's view of the subject, in three forms: the acute, the periodic, and the chronic. "The *acute* is the rarest of the three. We have seen it occur from hemorrhage in the puerperal state, in recovery from fevers, from excessive venereal indulgence, and in some forms of dyspepsia. The *periodic*, or paroxysmal, is much more frequent than the acute. This is often observed in individuals who have suffered from injuries of the head, females during pregnancy, at the catamenial period and afterwards, and in men whose brains are overworked. Like the form about to be mentioned, it is frequently hereditary, being derived from a parent predisposed to insanity, or addicted to intemperance. In such cases the probability of cure is very small." "Of all the forms of oinomania, the most common is the *chronic*. The causes of this are injuries of the head, diseases of the heart, hereditary predisposition, and intemperance. This is, by far, the most incurable form of the malady." During the paroxysm, the sufferer is "regardless," as Dr. Hutcheson says, "of his health, his life, and all that can make life dear to him." Dr. Hutcheson tells us, that he has only seen one case completely cured; and seclusion for two years was in this instance required.

*Mania*, the subject of section sixth of the long chapter sixth, is, perhaps, the more interesting and best recognized form of mental disease. Dr. Pritchard ranked it under intellectual insanity; by Dr. Tuke it is regarded as belonging primarily to the affective group. "While, therefore, we regard mania as usually having its origin in disordered emotions, we fully admit that the whole mind generally suffers in consequence, and that confusion then becomes universal throughout the 'countless chambers of the brain.'" Innumerable are the delusions which affect the course of thought and conduct pursued by the patient. Fury is not, as some suppose, identical with mania. As remarked by Esquirol, "If maniacs are more frequently furious than other insane persons, it must be attributed to their temperament, their extreme susceptibility, and the exaltation of all their faculties; circumstances which render them exceedingly impressible, and consequently very irritable and choleric." Among the symptoms of mania, some stress has been laid on an offensive emanation from the skin of the patient, which impresses the sense of smell of those entering his room, as would the odor of a mouse-trap. Even in acute mania we may speak of the varieties of *sthenic* and *asthenic.* "One patient may be mad from an excess, another from a deficiency of vital force. The one may require the lancet, the other, stimulants. Mania exhibits a considerable tendency to pass into dementia. General paralysis may supervene upon mania."

*Puerperal Insanity* is not the only form of mania which results from the condition of women produced by parturition. "On the contrary, melancholia, delusional forms of insanity, and even dementia, may ensue. The mortality from this disease is not large. Out of the ninety-two cases already referred to, only six died; and none within a period of less than six months after childbirth. Perfect recovery of the mental faculties follows in a large proportion of instances."

Chapter seventh is headed "*Statistics of Insanity,*" and in its first section we have an account of the "*Causes of Insanity.*" These are either *predisposing* or *exciting.* Under the first head comes hereditary predisposition, which is found in a third of the entire number of cases. Some make the proportion as high as one-half, or fifty per cent. As regards transmission by the two parents, the probability is greater on the side of the mother; and this is to be feared more with respect to the girls than the boys. That of the father, on the contrary, is more dangerous as regards the boys than the girls. As relates to the "*Relative Liability of the Sexes to Insanity,*" women, as taught early by Cœlius Aurelianus, are less subject to insanity than men—whether from less inherent susceptibility to the disease, or less exposure to the causes on the part of the former, we cannot say. The causative influence of age is shown in the greater liability to insanity in the decade between twenty and thirty years, according to Dr. Tuke, and between thirty and forty years, according to Esquirol. The summer months exhibit the greatest number of instances of attacks of insanity, and the minimum influence is in the winter ones. In contradiction to the inference which would be drawn from this fact, respecting the influence of climate, it is found that the disease is much more prevalent in cold than in temperate or warm countries. The writers on this point do not, as far as we are aware, take into consideration the element of race, in comparing the different peoples of cold and of warm climates. If we compare town with country life, on the score of their influence in relation to insanity, we shall find that the disease prevails, on the continent, more in towns than in the country, while in England an opposite state of things occurs. In speaking of occupation as productive of insanity, we should carry with us the opening remark of Dr. Tuke: "It is much to be regretted that any inference should be drawn from the absolute number of patients following particular occupations, instead of from the proportion to the population at large." The author introduces tables, the first of the kind constructed, in which is given the proportion of lunatics in asylums to the numbers engaged in various occupations in England and Wales. A question in which a lively interest will be felt by all classes is: Whether single or married life is most favorable to insanity? The conclusion arrived at is, that celibacy, as well as widowhood, may be regarded as a predisposing cause of insanity in both sexes; and it would seem that the married state is a greater preservative against insanity with men than with women.

"The Exciting Causes,—moral and physical,"—of insanity, come next under notice. It is a puzzling fact that, whereas the statistics furnished by M. Parchappe, and by Pinel and Esquirol, in France, and by the Bethlem Hospital in London, and the asylum at Ghent, make it appear that moral causes greatly preponderate, those of the York Retreat in England, and of the Irish asylums, and also of sixteen hospitals in the United States, indicate a majority on the side of physical causes. Among the moral ones, domestic troubles, or grief, stand first on the list in every return made; next in order is religious anxiety or excitement. "As regards physical causes, we observe that, with one exception, intemperance ranks first on the scale, while in the second, epilepsy, and disorders more or less connected with the uterus, are equally productive of insanity." Dr. Webster tells us, that fright and love give rise, each of them, to insanity in a a greater number of cases in women than in men.

Of *Recoveries*, the proportion ranges from 40 to 50 per cent. of those who have been treated in asylums. But, unhappily, the relapses are very numerous. Dr. Thurnam asserts that these "cannot be estimated as at all less than 50 per cent., or as one in every two cases discharged recovered." Dr. Tuke traced out the history of every patient who had been under care at the York Retreat during forty-four years, and he ascertained that 63·6 per cent. had subsequent attacks; that is to say, two out of every three cases of recovery from the first attack. As otherwise expressed, "in round numbers, then, of ten persons attacked by insanity, five recover, and five die, sooner or later, during the attack. Of the five that recover, not more than two remain well during the rest of their lives. The other three sustain subsequent attacks, during which at least two of them die." Notwithstanding this melancholy picture, we have the consolatory reflection, that, during long intervals of mental health, (in many cases of from ten to twenty years' duration,) the individual has lived in all the enjoyments of social life. The question has been often asked: "Is the

*mortality* greater among the insane than the sane?" "Undoubtedly it is." But this tendency to shorten life is not seen in the chronic form of the disease. It is only, or mainly so, in the acute stage.

Here terminates Dr. Tuke's share of the work before us. The remainder is written by Dr. Bucknill, and includes the chapter on the diagnosis, pathology, and treatment of insanity, and an appendix of cases. Our rapidly waning space, contracted as it is at the best, within very narrow and inadequate limits, makes it doubtful whether we shall be able to continue even the curt analysis with which we have treated Dr. Tuke's labor.

Chapter eighth is on the "*Diagnosis of Insanity.*" Dr. Bucknill points out the difficulties of the subject, and tells us that it presents itself to the physician either in a purely medical, or in a medico-legal point of view. According to the answer given in the first supposition, will depend, not only the medical treatment but the enjoyment or the loss of personal liberty. When the question is a medico-legal one, it may occur either in civil suits or proceedings, or in criminal trials. But in all these circumstances the ground of the diagnosis must be the same. Amid the difficulties in determining the question, the physician, called to see a patient who is supposed, or feared to be insane by his family or friends, will act wisely, as a preliminary measure, in making himself acquainted, as far as he possibly can, with the antecedents and history of the patient. In this investigation he must not, however, expect to be aided by receiving frank and full statements and explanations from the near relatives of the patient, who often seem bent on mystifying the whole subject—especially in denying the slightest taint of hereditary insanity in their family. The following considerations early press themselves on our attention, viz., *hereditary tendency, previous attacks, change of habits and disposition.* In reference to this last point, it must be remembered that it by no means follows, because a person has become "a changed man," he must, therefore, be an insane one. In estimating exaggerations or alterations of character, we must be careful to make allowance for those which take place naturally, and in healthy minds. Regard must be paid to the vagaries of hysteria, in taking account of sudden and remarkable changes of character. A careful inquiry into the habits of the patient will often enable the physician to discover an adequate cause for the production of insanity; as in the case, for example, of intemperance, which, when ascertained, will preclude the necessity of any further inquiry.

After these preliminary inquiries, the physician may now be supposed to be introduced to his patient, to whom he will be careful not to make any allusions respecting the mental disorder of the latter, but, instead, inquire after any ailment or bodily infirmity with which he may be afflicted. *Peculiarities of Residence and Dress* are worthy of attention. In the room occupied by the insane, things are generally found out of place. Articles of clothing are scattered around, everything is disarranged, and the dress and person of the patient often bears evident marks of want of care and cleanliness. Alone, these appearances are not entitled to much weight; otherwise they would condemn to seclusion a large class of habitual slovens and slatterns,—not to speak of college students and authors,—who are lamentably deficient in what phrenologists call the organ of order. There are seldom *Peculiarities of Bodily Condition* to aid us in the diagnosis of insanity. *Peculiarities of Voice and Gesture* are, on the other hand, often strongly expressive of mental disease, and they merit a careful study on the part of the physician. The *Physiognomy of Insanity* is also full of meaning; and it is handled with ability by the author. The principal characteristic in many of the patients laboring under mania, is the peculiar want of harmony in the expression of the features. The hair harsh and bristling, and the skin of the scalp becoming loose, are symptoms worthy of notice.

"The medical man should never omit to examine the ears. The discovery of a shrivelled ear tells an undeniable tale of profound mental disease. Altogether, the effect of mania, and, indeed, of all forms of insanity, is to stamp upon the patient a remarkable degree of ugliness; and there is no symptom of returning health more trustworthy and more pleasing than the restoration of personal beauty."

The physician passes from the observation of the signs of mania to the active investigation of the mental state, by questioning and conversing with the patient. The conversation should gradually be turned to induce the patient to speak about himself, and such topics chosen as will draw him out into an expression of his feelings, hopes and aspirations, crossed ambition, etc., and his plans; in describing which, the special hallucination or delirium will show itself. The means by which these results are obtained are clearly indicated by Dr. Bucknill. The diagnosis of mania had been preceded by that of dementia, and is followed by that of other forms of insanity—melancholia, monomania, the moral insanity of Pritchard, kleptomania, and loss of the muscular power ensuing—general paralysis. The *Detection of Feigned Insanity* is a leading point in the diagnosis of mental disease. "The most important diagnostic point of feigned insanity is the want of coherence of the manifestations, not only with mental diseases in general, but with the form or variety of insanity which is feigned in particular." If the patient can write, he should always be encouraged to do so. It has been observed that many of the insane who are backward in saying anything which would imply mental disorder, are quite expansive, and show their particular delusions on paper.

The chapter on the *Pathology of Insanity* evinces, on the part of the author, a suitable knowledge of the minute structure of the brain, and careful observations of the changes which it undergoes in the production of insanity. He does not, however, pretend to affirm, that a connection can always, or even often, be observed between obvious organic lesions and the functional changes which constitute mental disease; nor would he affirm that such a connection will ever be perceptible to our senses, although he believes that it does exist. The little cells of the vesicular neurine of the brain "are the agents of all that is called mind, of all our sensations, thoughts, and desires; and the growth and renovation of these cells are the most ultimate conditions of mind with which we are acquainted." And again; "Not a thrill of sensation can occur, not a flashing thought or a passing feeling can take place, without changes in the living organism; much less can diseased sensation, thought, or feeling occur without such changes—changes which we are not able to detect, and which we may never be able to demonstrate, but which we are, nevertheless, certain of." The following conclusion is reasoned out: viz. "that *diseased conditions which affect the mental functions must have their seat in the gray matter of the cerebral convolutions.*" As regards inflammation, we are told that, although it "may not be the actual condition of insanity, it is not unfrequently its cause." The explanation of this state of things is fully given by the author. Stress is laid by him on congestion of the cerebral convolutions as a common and sustaining cause of insanity. In "a congeries of organs," like the brain, there may be depression of function in one part and excitement of function in another. There is a difference also in the susceptibility of the different cerebral organs to be disturbed by morbid causes. The author, in these few sentences, uses, without perhaps his being aware of it, the very language of phrenology. The efficient causes of insanity are often defects of nutrition, as from loss of blood, impediments to healthy digestion and assimilation. "The commonest appearance met with in the brains of insane persons is that of shrinking;" measurements of the extent of which, in a large number of cases, are given in the present work. The author enumerates the apparent and exciting causes of the nutritive defect which results in cerebral atrophy and insanity. These are, 1. Poverty of blood; 2. Derangement of the ultimate structure between the nervous and vascular systems; 3. Molecular change effected by blows or violent concussions; 4. Consequences of inflammation and of frequent or long-continued congestion; 5. The most numerous class is that depending on want of rest. "Want of refreshing sleep we believe to be the true origin of insanity, dependent upon moral causes." Insanity by *sympathy* with lesions or irritations of other organs is noticed, and its mode of origin explained in this chapter. Next follows *an account of special pathological changes,* to which we are able merely to advert, while adding a remark by the author; that the most careful observations made on this subject, in France, are by M. Parchappe, Inspector-general of asylums in that country. Dr. Bucknill's summary notices of the Frenchman's labors are well arranged and selected, and make up, together with accompanying comments, the larger part of the chapter. "*Hypertrophy* of the brain is an interesting but rare form of pathological change. Pathological peculiarities in the thoracic and abdominal viscera are noticed. *Diseases of the heart and lungs* are common among the insane; and those of the *stomach* bear to insanity a relation of the highest importance. In acute melancholia, attended by refusal of food, the mucous membrane of this organ is frequently found to be inflamed, and softened or ulcerated. The *liver* does not suffer in an especial manner, and the *kidneys* are remarkably free from diseases in all the forms of insanity. The *reproductive organs* are frequently the seat of diseases, or of abnormal function. We cannot follow the author in his statements on the *Humoral Pathology of Insanity,* nor on those on the *Pathology of General Paralysis,* all of which are pertinent and suggestive.

The *Treatment of Insanity* is the subject of the tenth and last chapter of the volume. Dr. Bucknill begins with some general observations on the opinions of those who would not only give prominence to, but who would rely exclusively on, moral treatment in the sense in which they understand it. He next notices the principles of treatment laid down by a few authors, whose names stand highest in psychological medicine. First among these is Pinel, "the father of the modern treatment of insanity." Happily, when we read of the practice of beating the insane, and of other means of inspiring fear, it is as a portion of retrospective history, respecting whose records there is now one universal feeling of reprobation. Farther reference is made to the ameliorations of treatment brought about by Pinel, and based upon sound principles, and, in a great measure, completed by Esquirol. "The treatment of insanity may be considered under three heads—the Hygienic, the Moral, and the Medicinal." The *Medicinal Treatment,* the first discussed by the author, ought, he says, to be founded upon the ultimate diagnosis, which, as nearly as possible, refers the symptoms of each individual case to the exact pathological condition from which they arise. It may be divided into that of the acute and the chronic forms of the disease, into that the aim of which is curative, or which looks only to palliation; and in the former, into that which is adapted to the outbreak, and that to the more tranquil period of the disease. *Bleeding,* as a general practice, is not now had recourse to in the treatment of insanity. Perhaps, in this particular, we have passed from one extreme to another. The author tells us, that in the treatment of some two thousand cases he has never yet used the lancet. Preference is given, when abstraction of blood is indicated, to cupping and leeching. He "has, for many years, used *tartrate of antimony* in a certain class of cases of mania with the happiest results." The "benefit to be derived from it appears to bear a close relation to the tolerance which the patient has for it;" and hence, when its use is attended with nausea and depression he discontinues it. The cases best adapted to the use of the tartrate are of what may be called sthenic mania, occurring in full habits, with a strong pulse. *Calomel* ought not, as a rule, to be given in the treatment of mania, on account of the irritability of the nervous system which it tends to produce. Occasionally, as a purgative in combination with vegetable articles of this class, or as an alterative, it is serviceable. The right use of *opium* in the treatment of insanity is a question of paramount importance in psychological medicine. "At the present time the skillful and discriminating use of this drug may truly be called the sheet-anchor of the alienist* physician." Its dose, when the indications for its use are clear, ought to be large. It is inadmissible in mania so long as cerebral hyperæmia exists. Instructive reflections are made in reference to the complication of disease of the brain, such as coma and narcotism, in some cases in which opium has been used. We have seen such a complication in a fatal termination of delirium tremens. Dr. Bucknill believes that none of the narcotics, including "the new toy, Indian hemp," deserve a moment's consideration, in the treatment of mental disease, except opium and hyoscyamus. *Stimulants* and nourishment, in some cases of insanity, act like narcotics. As to *purgatives* and *aperients*, the author's opinion is, that a brisk purge is often more useful than any other remedy in the course of chronic and incurable insanity; but, as a means of curative treatment, active purgation is of little value. *Counter-irritation and derivation* are useful when employed in the right cases at the right time. Croton oil, rubbed on the shaven scalp, after the subsidence of acute symptoms and when the head is cool and there are no signs of plethora, is attended with excellent effects. *Tonics* suggest, of course, the use of quinine, which, dissolved in port-wine, often acts efficaciously in the latter stages of mania, in broken-down constitutions. *Bathing* is dwelt upon as among the most useful of the secondary remedies. The shower-bath, of not more than three minutes' duration, is occasionally useful in the intercurrent excitement of mania and of monomania: and as a tonic in irritably nervous and hysterical patients, and in sane melancholic ones.

---

* We object to this word here, so long, at least, as alien carries with it, in common speech and in written composition, a meaning of universal recognition, viz. a foreigner or a native of another country than of that in which he is a temporary resident. The Alien Office in England had to deal with aliens, (foreigners,) but not with insane persons. The chief and his subordinates in it might have been called alienists.

In more violent cases of cerebral hyperæmia, in acute cases of insanity, cold to the head alone is much more efficacious than the bath. The use of the warm-bath, either alone or in combination with cold to the head, is a most important remedy in the therapeutics of mental disease. The simple warm-bath allays irritation and promotes sleep. Here we would beg our readers not to confound, as is so often done, a warm with a tepid or a hot-bath, and to remember that the range of a warm-bath is between the limits of 92 and 98 degrees of Fahrenheit. The duration of the warm-bath will vary from half an hour to an hour.

A *résumé* of the principles upon which active remedies should be prescribed follows the notices of each of them separately. It evinces an observing and logical mind. We cannot do more now than merely to recommend a careful perusal of what is said by the author on the *Moral Treatment of the Insane,* which has been so often confounded with the physiological treatment. He quotes, in terms of approval, Dr. Conolly's recommendation of what may be called "an *individualized* treatment"—the beneficial exercise of a sane mind, happily constituted and well trained, on the insane mind. We quote but two sentences, out of pages, on the subject of moral treatment: "The most efficient method of loosening the hold of delusive opinion is by stimulating the exercise of healthy thought." "Argument is notoriously useless in the treatment of insane delusion." The subject of *Mechanical Restraint and Seclusion* is examined with brevity, but in such a manner as to leave no doubts of the views and settled practice of the author. These may be imparted to the reader in this single sentence: "We have for fourteen years conducted a large asylum, whose admissions during that time have amounted to eighteen hundred cases, without having had occasion to resort to the employment of mechanical restraint in the treatment of insanity."

A frontispiece to this volume exhibits "Types of Insanity," from photographs taken in the Devon County Lunatic Asylum; and in an Appendix we find a record of cases illustrated by these portraits.

In conclusion, we can say, in all sincerity, that seldom have the expectations raised by the language of the Preface been so fully realized in the execution of the work as they are in the volume of Drs. Bucknill and Tuke. Its publication removes all excuse that may, hitherto, have been alleged by students and practitioners of medicine for neglect of the subject, owing to the want of a work of insanity for ready reference—one which, like the present, contains sound principles, enforced and illustrated by a series of pertinent and well-digested facts, evidently accumulated by long personal experience, and much reading. Where we have found so much to commend, we may the less regret our inability, from want of space for the purpose, to interrupt our condensed synopsis by the language of criticism.

